# Horizontal Gene Acquisition of *Liberibacter* Plant Pathogens from a Bacteriome-Confined Endosymbiont of Their Psyllid Vector

**DOI:** 10.1371/journal.pone.0082612

**Published:** 2013-12-04

**Authors:** Atsushi Nakabachi, Naruo Nikoh, Kenshiro Oshima, Hiromitsu Inoue, Moriya Ohkuma, Yuichi Hongoh, Shin-ya Miyagishima, Masahira Hattori, Takema Fukatsu

**Affiliations:** 1 Electronics-Inspired Interdisciplinary Research Institute (EIIRIS), Toyohashi University of Technology, Toyohashi, Japan; 2 Japan Collection of Microorganisms, RIKEN BioResource Center, Tsukuba, Japan; 3 Department of Liberal Arts, The Open University of Japan, Chiba, Japan; 4 Graduate School of Frontier Sciences, University of Tokyo, Kashiwa, Japan; 5 Citrus Research Division, National Institute of Fruit Tree Science, Kuchinotsu, Japan; 6 Graduate School of Bioscience and Biotechnology, Tokyo Institute of Technology, Tokyo, Japan; 7 Center for Frontier Research, National Institute of Genetics, Mishima, Japan; 8 Bioproduction Research Institute, National Institute of Advanced Industrial Science and Technology (AIST), Tsukuba, Japan; University College Dublin, Ireland

## Abstract

he Asian citrus psyllid *Diaphorina citri* is a notorious agricultural pest that transmits the phloem-inhabiting alphaproteobacterial ‘*Candidatus* Liberibacter asiaticus’ and allied plant pathogens, which cause the devastating citrus disease called Huanglongbing or greening disease. *D. citri* harbors two distinct bacterial mutualists in the symbiotic organ called bacteriome: the betaproteobacterium ‘*Candidatus* Profftella armatura’ in the syncytial cytoplasm at the center of the bacteriome, and the gammaproteobacterium ‘*Candidatus* Carsonella ruddii’ in uninucleate bacteriocytes. Here we report that a putative amino acid transporter LysE of *Profftella* forms a highly supported clade with proteins of *L. asiaticus*, *L. americanus*, and *L. solanacearum*. *L. crescens*, the most basal *Liberibacter* lineage currently known, lacked the corresponding gene. The *Profftella-Liberibacter* subclade of LysE formed a clade with proteins from betaproteobacteria of the order Burkholderiales, to which *Profftella* belongs. This phylogenetic pattern favors the hypothesis that the *Liberibacter* lineage acquired the gene from the *Profftella* lineage via horizontal gene transfer (HGT) after *L. crescens* diverged from other *Liberibacter* lineages. *K*_A_/*K*_S_ analyses further supported the hypothesis that the genes encoded in the *Liberibacter* genomes are functional. These findings highlight the possible evolutionary importance of HGT between plant pathogens and their insect vector’s symbionts that are confined in the symbiotic organ and seemingly sequestered from external microbial populations.

## Introduction

The Asian citrus psyllid *Diaphorina citri* (Hemiptera: Psyllidae) is an important agricultural pest that transmits a serious citrus disease, Huanglongbing (HLB) or greening disease. This insect is widely distributed in Asia, and is spreading into other citrus growing regions worldwide [1]. The causative agents of HLB are considered to be three species of a fastidious phloem-inhabiting alphaproteobacterial lineage of the genus *Candidatus* Liberibacter: *L. asiaticus*, *L. americanus*, and *L. africanus* [2], [3]. *D. citri* vectors *L. asiaticus* and *L. americanus* in Asia and the Americas, and the African citrus psyllid *Trioza erytreae* (Hemiptera: Triozidae) vectors *L. africanus* in Africa [1], [2], [3], [4]. Similar diseases have been found in potatoes, tomatoes and other solanaceous crops infected with *L. solanacearum* (also known as *L. psyllaurous*) [5]. These *Liberibacter* species are very fastidious, but *L. crescens*, the species recovered from mountain papaya, has recently been reported to be readily culturable [6]. Complete genome sequences have been determined for *L. asiaticus* [7], *L. solanacearum* [5], and *L. crescens* [6], whereas draft genome sequence is available for *L. americanus* [8].

In its abdomen, *D. citri* possesses a large yellow symbiotic organ called the bacteriome, where two distinct symbionts are harbored [9]. The betaproteobacterium ‘*Candidatus* Profftella armatura’ is located in the syncytial cytoplasm at the center of the bacteriome, whilst the gammaproteobacterium ‘*Candidatus* Carsonella ruddii’ is found in uninucleate bacteriocytes on the surface of the bacteriome. Our previous study revealed that *Profftella* is a toxin-producing defensive symbiont that potentially protects *D. citri* from natural enemies, while *Carsonella*_DC is a nutritional symbiont that provides the host with essential amino acids, which are scarce in the psyllid’s diet of phloem sap [10].

Here we report that the *Liberibacter* lineage horizontally acquired a putative transporter gene from a bacterium closely related to the extant *Profftella*.

## Materials and Methods

HGT candidates in the *Profftella* genome were extracted by BLASTP searches [11] against NCBI nr database, using deduced amino acid sequences of all protein coding genes on the *Profftella* genome as queries. Amino acid sequences were aligned using MAFFT 6.847 [12], followed by manual refinement. Amino acid sites corresponding to alignment gap(s) were omitted from the data set. The best fitting amino acid substitution model for the alignment was estimated using ProtTest3 [13]. For the present analysis, ProtTest selected LG with a gamma distribution (+G), a proportion of invariable sites (+I) and empirical base frequencies (+F) as the best fitting substitution model, followed by WAG with the options +I +G +F. Phylogenetic trees were inferred by the Maximum Likelihood (ML) [14] and the Bayesian Inference (BI) [15] methods. ML trees were constructed using RAxML7.2.1 [16] with LG + G + I + F model. The support values for the internal nodes were inferred by 1,000 bootstrap replicates. In the BI, we used the program MrBayes 3.1.2 [15]. Since the LG model is not implemented in MrBayes, WAG as the next best available model was used with the options +I +G +F. In total, 18,000 trees were obtained (Nruns  =  2, Ngen = 900000, Samplefreq = 100), and the first 2,000 of each run were considered as the “burn in” and discarded. The posterior probability of each node was used as the support value of the node. We checked that the potential scale reduction factor was approximately 1.00 for all parameters and that the average standard deviation of split frequencies converged towards zero.

*K*_S_ and *K*_A_ values were calculated as described previously [17]. Statistical significance of the obtained *K*_A_/*K*_S_ value was tested against a bootstrap distribution of *K*_A_/*K*_S_ values, which was generated by 10,000 bootstrap resamplings of codons from the original alignment. When *K*_S_ values calculated from resampled alignments were close to saturation values (larger than 2.0 per site), the *K*_S_ values was set as 2.0 for the estimation of *K*_A_/*K*_S_ value.

To analyze the structural organization, the genomic sequences of *L. asiaticus* str. psy62 [accession no. NC_012985], *L. asiaticus* str. gxpsy [NC_020549], *L*. *solanacearum* CLso-ZC1 [NC_014774], *L*. *americanus* PW_SP [AOFG01000001-22], and *L*. *crescens* BT-1 [NC_019907] were obtained from GenBank.

## Results

**Figure 1:**
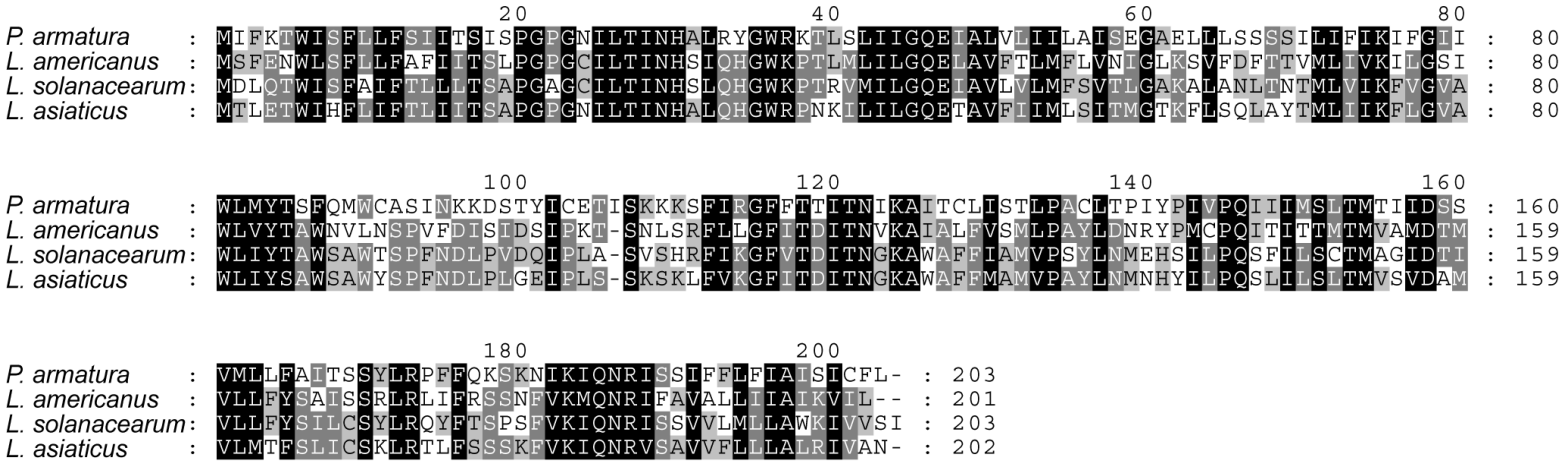
Alignment of amino acid sequences of LysEs. Residues conserved in all lineages, three lineages, and two lineages are shaded black, dark gray, and light gray, respectively.

BLASTP searches against the NCBI nr database demonstrated that the putative LysE protein (accession no: YP_008343788) of *Profftella* is significantly similar to its counterparts of *Liberibacter* spp. The top BLAST hit was the “putative homoserine/homoserine lactone efflux protein” of *L. asiaticus* str. psy62 and str. gxpsy (accession nos: YP_003065395 and YP_007599438, respectively. E  =  2e-47 for the both strains). Subordinate hits were “putative homoserine/homoserine lactone efflux protein” of *L. americanus* (WP_007557425, E  =  2e-43) and *L. solanacearum* (YP_004063007, E  =  3e-42), followed by LysE superfamily proteins, such as “lysine transporter LysE”, “threonine transporter RhtB”, and “homoserine/homoserine lactone efflux protein”, of various betaproteobacterial species belonging to the order Burkholderiales. Putative orthologs of the *Profftella lysE* were observed in *L. asiaticus*, *L. americanus*, and *L. solanacearum*, but not in *L. crescens,* the most basal *Liberibacter* lineage currently known [6]. The LysE of *Profftella* was 40–43% identical to its orthologs of *Liberibacter* spp (Fig. 1). No other HGT candidates were found between *Profftella* and *Liberibacter* spp.

**Figure 2:**
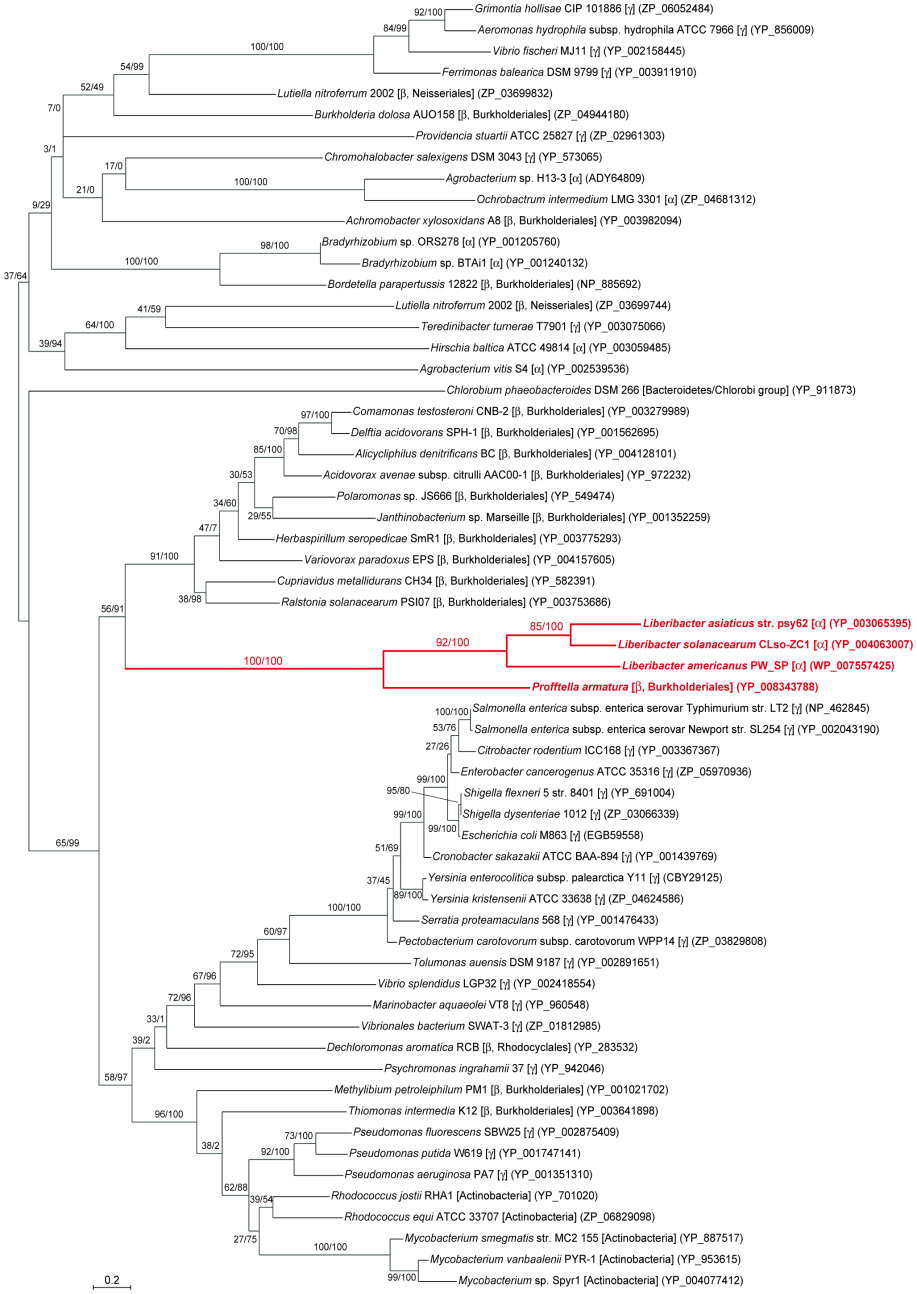
Phylogenetic position of *Profftella* LysE in related transporter proteins. A total of 185 unambiguously aligned amino acid sites were subjected to the analysis. A maximum likelihood phylogeny is shown, whereas a Bayesian analysis inferred essentially the same result. On each node, support values of maximum-likelihood analysis/Bayesian posterior probabilities are shown. Scale bar indicates substitutions per site. Source organisms are shown with higher bacterial taxa in brackets. α, β, and γ indicate classes of the *Proteobacteria*, respectively. Accession numbers of proteins are shown in parentheses. The *Profftella-Liberibacter* cluster is highlighted in red.

**Figure 3:**
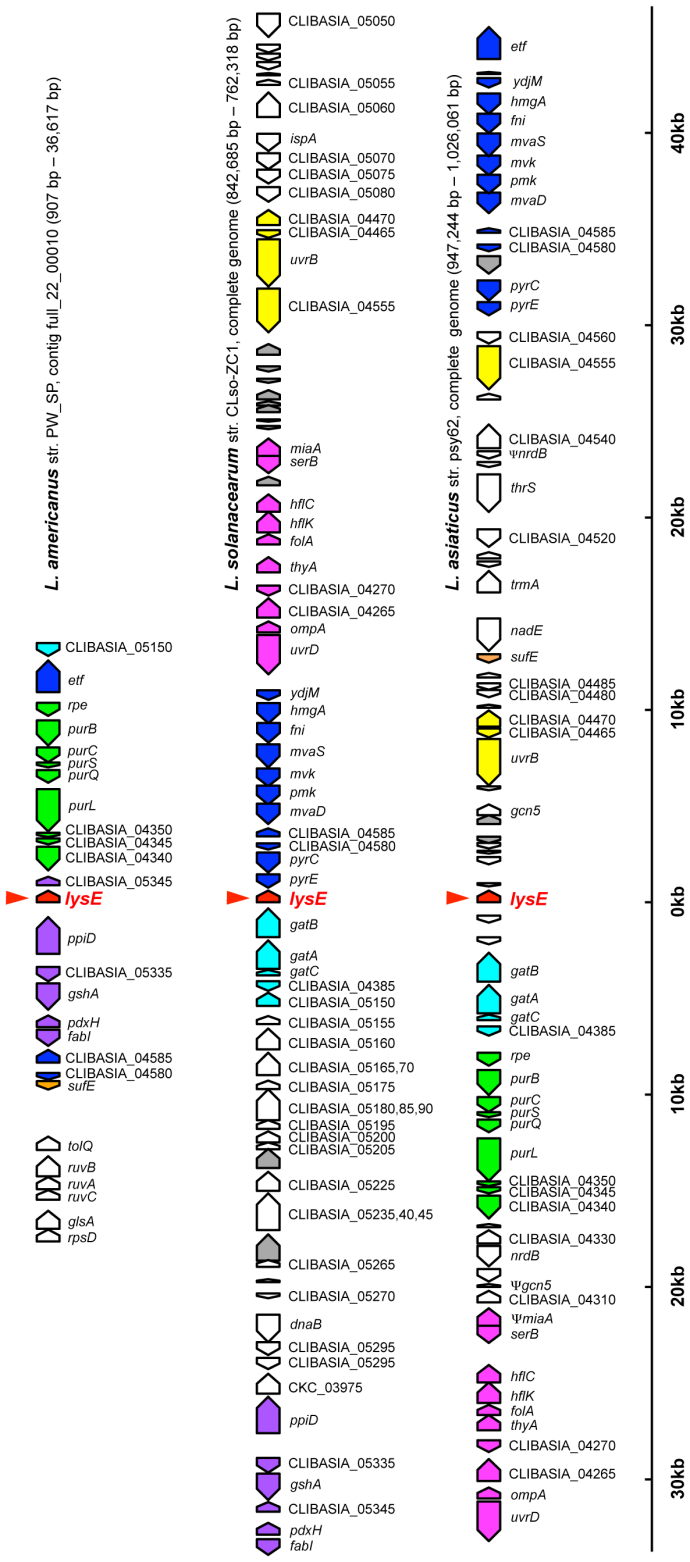
Gene order of *lysE* flanking regions in the *Liberibacter* genomes. Pentagons indicate genes with coding directions. Colored pentagons are conserved among the three *Liberibacter* genomes. Genes with the same color indicate gene clusters. *lysE*-type protein genes are indicated by arrowheads. Gray pentagons are phage related genes. For *L. asiaticus*, structural organization of the str. psy 62 genome is shown, whereas that of str. gxpsy was essentially the same.

Molecular phylogenetic analysis demonstrated that the LysE of *Profftella* forms a highly supported clade with the proteins of *Liberibacter* spp. (Fig. 2). The *Profftella-Liberibacter* subclade was placed within a clade that largely consisted of the LysE sequences of betaproteobacteria and gammaproteobacteria that are paraphyletic to *Betaproteobacteria* [18]. Moreover, this subclade formed a clade with proteins from betaproteobacteria of the order Burkholderiales, to which *Profftella* belongs [19]. This phylogenetic pattern, together with the presence/absence of the orthologous genes in *Liberibacter* spp., is most simply explained by the hypothesis that the *Liberibacter* lineage acquired the transporter gene from the *Profftella* lineage via horizontal gene transfer (HGT) after *L. crescens* diverged from other *Liberibacter* lineages. The structural organizations of the *lysE* flanking regions were partially conserved among genomes of *L. asiaticus*, *L. americanus* and *L. solanacearum* (Fig. 3), which were all assembled *de novo* without reference to one another [5], [6], [7], [8], further supporting a single acquisition of this gene in the *Liberibacter* lineage.

The *K*_A_/*K*_S_ ratio between *lysE* genes of *L. asiaticus* and *L. solanacearum* was significantly lower than 1 (*K*_A_ = 0.24, *K*_S_ = 1.61, *K*_A_/*K*_S_ = 0.15, *p* < 0.0001). Whereas the *K*_S_ values both between *L. asiaticus* and *L. americanus* and between *L. solanacearum* and *L. americanus* were saturated (> 3.00), the *K*_A_ values were still as low as 0.42 and 0.39, respectively. These results support the hypothesis that the *lysE* genes of *Liberibacter* spp. are under purifying selection and thus are functional.

## Discussion

The present study demonstrated that the *Liberibacter* lineage horizontally acquired a *lysE*-type transporter gene from the *Profftella* lineage, an endosymbiont of their vector insect. *K*_A_/*K*_S_ analyses further supported the hypothesis that the genes encoded in the *Liberibacter* genomes are functional. Although their true functions are yet to be identified, LysE superfamily proteins of various bacteria are generally involved in exporting substrates, playing important roles in resistance to toxic substances, in maintenance of optimum intracellular concentration of metabolites, and in excretion of regulatory molecules [20], [21]. Thus, it is probable that *Liberibacter* have acquired novel functions through this HGT. Whereas HGTs are rampant among bacteria [22], [23], such transfers of genes are rare in intracellular bacteria that are harbored in insects’ symbiotic organ and are seemingly sequestered from external microbial populations [24], [25], [26]. Apparently, *Profftella*, the putative donor lineage of the *lysE* gene, is this type of endosymbiont. In this context, infection style of *Liberibacter,* the putative accepter of the gene, would be noticeable. As *Liberibacter* spp. are transmitted by psyllids in a persistent manner, exhibiting near systemic infection of various organs and tissues [27], they may also intrude into the bacteriome of the vector psyllids, having opportunity of HGT with endosymbionts therein. The present findings highlight the previously unrecognized possible evolutionary importance of HGT between plant pathogens and their vector’s mutualists that are confined in symbiotic organs.
